# Characterization of phi112, a Molecular Marker Tightly Linked to the *o2* Gene of Maize, and Its Utilization in Multiplex PCR for Differentiating Normal Maize from QPM

**DOI:** 10.3390/genes14020531

**Published:** 2023-02-20

**Authors:** Alla Singh, Chikkappa Karjagi, Sehgeet Kaur, Gagan Jeet, Deepak Bhamare, Sonu Gupta, Sunil Kumar, Abhijit Das, Mamta Gupta, D. P. Chaudhary, Bharat Bhushan, B. S. Jat, Ramesh Kumar, M. C. Dagla, Manoj Kumar

**Affiliations:** 1 ICAR-Indian Institute of Maize Research, P.A.U. Campus, Ludhiana 141004, India; 2 ICAR-Indian Institute of Maize Research, Pusa Campus, Delhi 110012, India; 3 School of Agricultural Biotechnology, Punjab Agricultural University, Ludhiana 141004, India; 4 ICAR—Central Institute for Research on Cotton Technology, Mumbai 400019, India

**Keywords:** Quality Protein Maize, phi112, amino acid metabolism, protein-DNA interactions, biofortification, nutrition

## Abstract

Quality Protein Maize (QPM) contains higher amounts of essential amino acids lysine and tryptophan. The QPM phenotype is based on regulating zein protein synthesis by *opaque2* transcription factor. Many gene modifiers act to optimize the amino acid content and agronomic performance. An SSR marker, phi112, is present upstream of the *opaque2* DNA gene. Its analysis has shown the presence of transcription factor activity. The functional associations of opaque2 have been determined. The putative transcription factor binding at phi112 marked DNA was identified through computational analysis. The present study is a step towards understanding the intricate network of molecular interactions that fine-tune the QPM genotype to influence maize protein quality. In addition, a multiplex PCR assay for differentiation of QPM from normal maize is shown, which can be used for Quality Control at various stages of the QPM value chain.

## 1. Introduction

Maize (*Zea mays* L.) is an important global crop that finds applications in various sectors, including food, feed and energy [1,2,3]. Maize is considered a potential solution for crop diversification of rice-based cropping systems to bring ecological sustainability into farming models. Cereal crops are the only source of nutrition for the vast majority of the world’s population. In South-East Asia and Sub-Saharan Africa, the most important grains are wheat, rice, and maize. Cereals have a low protein content. As a result, communities that rely primarily on grains for their nutritional requirements suffer from protein deficiency. Maize contributes around 15% to global protein consumption; however, maize proteins have a low nutritional value [4]. Maize proteins are deficient in essential amino acids such as lysine, tryptophan, and methionine. The main proteins in maize grain include zeins and glutelins, together with relatively small quantities of albumins and globulins. Zein proteins are involved in the formation of protein bodies in the endosperm of maize seeds. These protein bodies provide hardness to the endosperm, resulting in hard flint and dent grains, which are liked by farmers due to excellent post-harvest storage properties. Zein proteins are divided into four classes based on their function: α, β, γ and δ. Zein proteins do not contain any lysine, with the exception of δ-zeins, which contains one lysine codon. In the 1920s, a naturally occurring mutant of maize, *opaque-2*, was found in the USA that had soft endosperm [5]. In 1961, it was found that homozygous *o2* maize has higher levels of lysine and tryptophan [6]. Since then, many more mutants have been found such as floury-2, Mucronate, Defective Endosperm 30, etc., but opaque-2 was taken further with great enthusiasm. The increase in protein quality that includes high lysine and tryptophan content resulted from an increase in the proportion of non-zein proteins concomitant with a decrease in the proportion of zein proteins in the opaque-2 endosperm. However, selection for maize mutants containing high lysine and high tryptophan content has resulted in negative pleiotropic effects, of which the most significant is the formation of a soft maize endosperm, prone to various diseases and poor yield and agronomic performance. In *opaque* mutants of maize, the *o2* function is compromised, leading to less zein synthesis, especially α-zein synthesis, followed by simultaneous synthesis of higher nutritional quality non-zein proteins that raise the nutritional status. The absence of particular zein proteins leads to smaller protein bodies, thus affecting the starch packing, which together leads to soft kernels and renders the kernel prone to post-harvest infections.

The discovery of the nutritional superiority of *opaque2* (*o2*) maize mutants due to elevated levels of lysine and tryptophan has opened a new opportunity to improve the cereal protein quality [7]. Since then, extensive studies on *opaque2* (*O2*) gene have led to an understanding of the genetic, molecular and biochemical basis of the *O2* gene. One of the finest outcomes of the several efforts directed towards the improvement of protein quality in maize is the hard endosperm *o2* genotypes, most commonly referred to as Quality Protein Maize (QPM) [8]. The nutritional superiority of QPM over normal wild-type maize can alleviate childhood malnutrition in maize-consuming, low-income countries. Apart from higher protein quality, based on sensory characteristics also, QPM has good consumer acceptance and, in some cases, is preferred over normal maize [9]. The QPM phenotype (increased lysine and tryptophan coupled change with change in kernel traits) is controlled by the molecular function of *o2* transcription factor. It regulates the synthesis of zein proteins, which impart hardness to the maize kernel and several other genes involved in protein folding, stress response, etc.

To solve the problems associated with *opaque-2* maize, research efforts were pioneered at CIMMYT. A conservative approach was followed to maintain protein and grain quality simultaneously. The work was undertaken on two genetic systems, opaque-2 and endosperm modifier genes. Focus was directed on selecting endosperm modifications in opaque-2 maize to restore grain quality with the help of plant breeding. The new, improved varieties of opaque-2 were called Quality Protein Maize (QPM). In QPM, the increased amino acid content and kernel hardness are in balance, offering higher nutrition and good agronomic performance. Thereafter, quantitative trait loci associated with endosperm modification were located and molecular mechanisms behind improved protein quality were deciphered [10,11]. Protocols for the assessment of grain quality and the progress of breeding efforts were standardized. The endosperm modifiers were found to primarily influence the synthesis and spatial distribution of α-zein [12]. Holding [13] studied the organization of zein proteins at different stages of protein body formation in the endosperm. Li et al. [14] revealed structural variations in the genome of a South African QPM line K0326Y through long-read sequencing. Molecular studies to characterize the genetic systems concerning protein quality have been carried out in the last decade. Hunter and coworkers performed a microarray analysis of the changes brought about by the opaque-2 mutations [15]. They have reported alterations in gene regulation of around 236 genes. These genes span various pathways and cell structures, such as amino acid and carbohydrate metabolism, cytoskeleton, membrane transport, protein turnover and oil metabolism, etc. Many factors involved in transcription and translation, molecular chaperones, signal transduction and secondary metabolism have also been reported in the above microarray analysis. Li et al. [14] have provided a comprehensive overview of the cellular changes during QPM development. The authors demonstrated that ATP availability is an important parameter in endosperm development. Inside the maize endosperm, glycolysis is the main pathway for the generation of ATP. Normal functioning of starch biosynthesis, normal zein function, endoplasmic reticulum and ATP availability result in vitreous endosperm. However, in *o2*, the above functions are disrupted. The enzymatic activity of Pyruvate Phosphate dikinase is reduced, which affects glycolysis and limits the availability of ATP. Together with this, starch biosynthesis activity also reduces. This impacts the complexation of starch and protein bodies, resulting in soft endosperm in *o2* maize. In QPM, the starch biosynthesis and glycolysis pathway are rescued, leading to a relatively hard kernel with improved amino acid characteristics. Gibbon and Larkins [16] have reviewed molecular genetic approaches for improvement in maize protein quality. Zhan et al. [17] analyzed the 186 putative direct and 1677 indirect targets of O2 transcription activity. These include genes involved in nutrient reservoir activity, transcription and translation-related functions, protein serine/threonine kinase activation and protein phosphorylation.

Transgenic techniques have also been used to improve maize protein quality. Monsanto developed the LY038 transgenic maize line, which has higher amounts of lysine in the grain. This maize line contains a dihydrodipicolinate synthase enzyme (DHDPS) derived from Corynebacterium glutamicum. The bacterial DHDPS has reduced feedback inhibition in lysine synthesis, allowing lysine to accumulate in the cellular amino acid pool. Huang and team [18] have used an RNAi approach to reduce the levels of α-zeins, leading to an increase in lysine and tryptophan content. The porcine α-lactalbumin was used to increase protein quality in maize [19]. Yu et al. [20] have used a high lysine-rich protein, sb401, from potato to make maize transgenics. The transgenics displayed higher proportions of lysine and tryptophan. Yue et al. [21] used cotton protein GhLRP, Chang et al. [22] used AtMAP18 of Arabidopsis thaliana and Liu et al. [23] used microtubule-associated protein SBgLR to enhance maize protein quality. The agronomic performance of transgenic maize was similar to normal maize. Wang et al. [24] transformed SBgLR from potato and TSRF1 from tomato to make transgenic maize that displayed increased nutritive quality and resistance to salt stress. However, QPM is a naturally biofortified product and hence, its reach covers non-GM areas as well. In addition, it is a potential candidate for hidden hunger eradication in many parts of the world, where maize is already a staple diet.

Several QPM hybrids have been released commercially. However, it is realized that commercialization of QPM produce requires its rapid differentiation from normal maize. Many physiological and biochemical processes influence the plant type and traits during development and senescence [25,26]. Rapid detection protocols have been designed for several traits in plants, including amylose [27], photosynthetic parameters [28], plant viruses [29], prediction of nitrogen use efficiency [30], etc. The protein-level differences of QPM from normal maize have been elucidated [31,32]. An optimized protocol for measuring the protein quality of maize was described [33], which measures tryptophan and protein in a shorter period. To enable commercialization, it is envisaged that an alternative test that supplements the protocol described by [33] will be useful. The advances in understanding the molecular basis of the *o2* gene have identified the key sequence differences in wild-type genes and the molecular markers that co-segregate with the *o2* gene. Three molecular markers that differentiate QPM (*o2*) from normal maize (*O2*) are umc1066, phi057 and phi112. These are sequence-tagged microsatellite (STMS) markers. Out of the three markers, umc1066 and phi057 are codominant, and phi112 is dominant (present in normal and absent in QPM/opaque genotypes). The presence/absence variation of phi112 can be exploited to fabricate molecular tests for differentiating QPM from normal maize. The gross polymorphism, in the form of presence/absence variation, makes phi112 an interesting candidate for further analysis. In the present study, the phi112 marked DNA in maize was characterized and used to determine the genetic nature of one of the differences between QPM and normal maize. This study also shows the computational functional characterization of the DNA and its putatively interacting protein. This is important to decipher the intricate molecular network that governs the QPM phenotype and maintains a balance between nutrition and agronomy in maize. Its understanding is essential to devise breeding strategies based on gene modifier function, along with introgression of *opaque2* alleles in elite maize germplasm.

Since maize is a cross-pollinated crop and protein quality mediated by o2 is a recessive trait, it is important to conduct Quality Control of the QPM produce. The development of a genetic differentiation assay is expected to help segregate QPM from normal maize, thus enabling higher remuneration of the QPM produce and objectivity in various steps of the value chain. A multiplex PCR assay based on phi112 has been developed to differentiate QPM from normal maize in the present study.

## 2. Materials and Methods

### 2.1. Sequence Retrieval

The sequences of *Zea mays opaque2* and prolamine-box binding factor (PBF1) transcription factors were retrieved from the National Center of Biotechnology Information (NCBI). Basic local alignment was performed using the BLASTN module. Forward and Reverse primer sequences for phi112 STMS were taken from the MaizeGDB database [34]. The expression profiles of Pbf1 and Dof1 were also taken from the MaizeGDB database.

### 2.2. DNA Motif Discovery

The motifs in phi112 marked DNA were predicted using the MEME suite of search algorithms [35,36]. Gene ontology identification was performed using the *Arabidopsis thaliana* (Plant) database through the Gene Ontology for Motifs (GOMo) module.

### 2.3. Protein Interaction Analysis

Functional associations in the form of interacting proteins were identified using the STRING web server [37].

### 2.4. Generation of a Homology Model of Zea Mays PBF1

The homology model of *Zea mays* PBF1 transcription factor was generated using the SWISS-MODEL program [38].

### 2.5. Elucidation of Protein–DNA Interaction

The interaction of the PBF1 partial sequence with target DNA was elucidated using a hybrid algorithm of template-based modeling and *ab initio-free* docking, employed in the HDOCK program [39,40]. Polar contacts between protein and DNA were determined using the PyMol program (The PyMOL Molecular Graphics System, Version 1.2r3pre, Schrödinger, LLC).

### 2.6. PCR Amplification

The DNA of normal maize line MIL 10-378 and QPM line MIL 10–63 was extracted by the cetyltrimethylammonium bromide method [41]. A total of 1 µL of two concentrations of genomic DNA, *viz*., 10 and 20 ng/µL, each of normal and QPM genomic DNA, were used in a 20 µL PCR for amplification using 2X premix containing Ta Polymerase, dNTP mixture and buffer. The primers phi112 and phi217698 were used individually and in combination in multiplex reactions on normal maize and QPM DNA. The PCR cycle conditions for amplification were as follows: denaturation at 95 °C, annealing at 58 °C and extension at 72 °C, for a total of 30 cycles. The PCR product was loaded on 2% agarose gel and visualized using Gel Documentation System.

## 3. Results and Discussion

**Figure 1:**
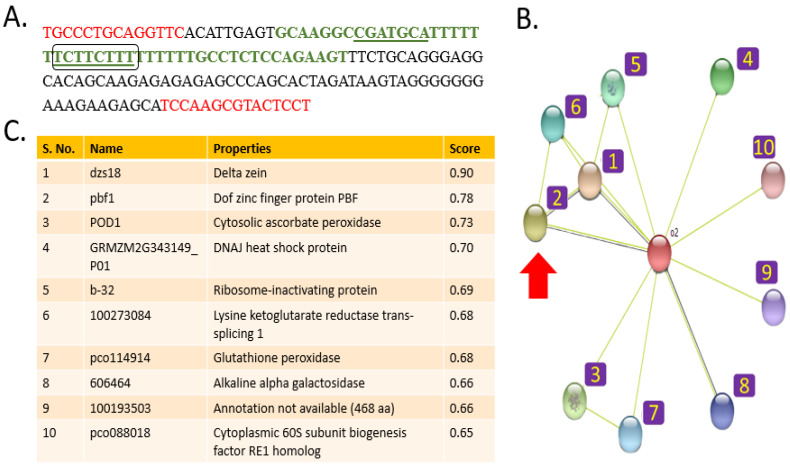
phi112 marked DNA and *opaque2* functional associations. (**A**). ph112 marked DNA. The primer sequences are shown in red. The discovered motifs are underlined. The motif discovery associated with transcription factor activity is boxed. The green region marks the DNA sequence used to perform protein–DNA interaction analysis. (**B**). Functional associations of opaque2 protein (numbers denote proteins mentioned in the Table). PBF1 transcription factor is marked with an arrow. (**C**). List of interacting proteins with the score indicating the association’s likelihood.

The presence/absence variation provided by the phi112 marker, which differentiates normal maize from QPM, makes it a good candidate for designing molecular tests to understand the underlying mechanism. Being close to the *opaque2* gene, a transcription factor involved in the QPM phenotype, analysis of phi112 marked DNA is important to determine the mechanism of its influence over maize phenotype. Using phi112 Forward and Reverse sequence, the intervening DNA sequence was retrieved using *Zea mays* genome (taxid: 4577). This intervening DNA, along with phi112 primer sequences, was termed ‘phi112 marked DNA’, which is 152 base pairs long (Figure 1A). The phi112-marked DNA was scanned for DNA motifs using the MEME Motif Discovery module [33,34]. Three motifs were discovered, out of which Gene Ontology identification using the *Arabidopsis thaliana* (Plant) database revealed transcription factor activity in one motif TCTTCTTT (shown in green in Figure 1A). This indicated that TCTTCTTT could be one of the regions associated with a transcription factor. However, as the discovered motif was identified based on the *A. thaliana* database, it was necessary to confirm if it also functions in maize. Thus, interaction analysis of the motif-containing DNA with its putative transcription factor protein is desired.

To find the putative transcription factor of the tightly regulated opaque2 system, functional associations of the opaque2 transcription factor were elucidated using the STRING database (Figure 1B). The database predicts functional associations based on experimental techniques such as co-expression or text-mining. A transcription factor, PBF1, was found to be functionally associated with opaque2 with a high probability (score = 0.780) as shown in Figure 1C. PBF1 is also associated with proteins such as δ-zein and ribosome-inactivating protein b-32 (Figure 1B).

**Figure 2:**
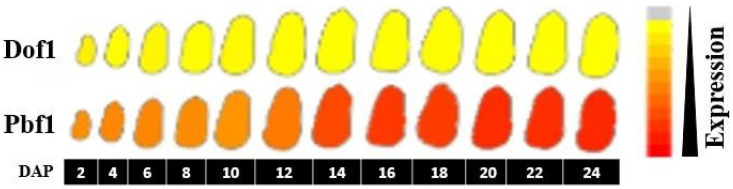
Expression profile of Dof1 and Pbf1 genes. Gene expression of Dof1 and Pbf1 in seed at various Days after Pollination (DAP) is depicted, data being retrieved from the MaizeGDB database. The expression scale is shown on the right, with dark red indicating the highest expression.

PBF1 is a prolamin box-binding factor-1 protein. It has been found to bind to P-box DNA sequences in the promoter regions of 19 kDa and 22 kDa zein proteins [42]. It also binds to 27 kDa zein protein, albeit with lower affinity. In the later stages of seed development, PBF1 is thought to interact with other proteins and has been proposed as a ‘recruiter’ of the Opaque2 transcription factor [42]. We wanted to ascertain if PBF1 can bind to the transcription factor associated motif in phi112-marked DNA. Apart from the PBF1 protein, a class of proteins referred to as Dof, containing zinc-finger motifs, has been found to bind the Pbox region of zeins [43]. Dof-domain proteins play a role in transcription activation or repression in diverse plant phenomena [44]. However, the STRING server results denote the Pbf1 gene as Dof zinc finger protein PBF. It has been shown that PBF1 does not contain Dof motifs as it does not bind zinc [42]. To remove any ambiguity, we searched the MaizeGDB database for expression profiling of Dof1 and PBF1 proteins (Figure 2). Therefore, we used the Pbf1 sequence for further DNA–protein interaction analysis.

**Figure 3:**
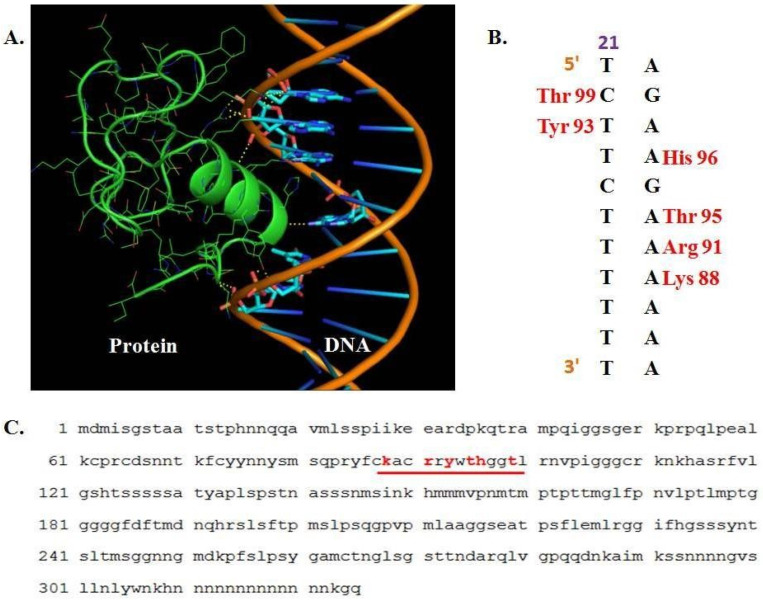
DNA–protein interaction. **(A**). PBF1 partial structure (green) interacting with DNA (orange double helix with nucleotides in cyan). (**B**). Nucleotides and amino acids involved in the interaction. Nucleotides and amino acids are shown in one- and three-letter codes. 5′ and 3′ ends of the sense strand of DNA have been marked. (**C**). The region of PBF1 that binds phi112 marked DNA has been underlined in red. The amino acids that make polar contact with DNA have been highlighted in red.

To further characterize the PBF1 protein, it was modeled via an automated homology algorithm. It showed a 17.39% sequence identity with endothelial transcription factor GATA-2, deposited as 509B entry in Protein Data Bank [45]. The structure has a Global Model Quality Estimation (GMQE) and QMEAN score of 0.34 and −3.98, respectively. Though the modeled structure is partial, its identity with transcription factor confirmed the possibility of DNA binding activity. To find the possibility of *Zea mays* PBF1 binding to the phi112 marked DNA and to elucidate the potential binding site in DNA, a longer sequence of 48 nucleotides, spanning 20 nucleotides on the sides of TCTTCTTT DNA sequence (marked in yellow in Figure 1A) was taken further for analysis. The PBF1–DNA interaction was analyzed via a hybrid docking algorithm utilizing template-based modeling and ab initio free docking (Figure 3A). It was found that PBF1 binds partially with the computationally discovered motif TCTTCTTT. The DNA motif that interacts with PBF1 is CTTCTTT (Figure 3B). Both the sense and antisense strand nucleotides interact with amino acids in PBF1. The region of the PBF1 transcription factor that interacts with DNA is a helix, and its position is shown in Figure 3C. The protein motif that interacts with DNA is Lys-Ala-Cys-Arg-Arg-TyrTrp-Thr-His-Gly-Gly-Leu (amino acids). Tyrosine 93 and Threonine 99 interact with the sense strand of DNA, while Lysine 81, Arginine 91, Threonine 95 and Histidine 96 interact with nucleotides on the antisense DNA strand. 

The use of a longer DNA sequence as a query for DNA–protein interaction and the localization of interaction in the discovered motif indicate that the PBF1 transcription factor can bind the phi112 marked DNA at motif CTTCTTT. Therefore, phi112 marked DNA has transcription factor activity. The presence/absence variation of phi112 marked DNA implicates its strong role in determining the normal versus QPM phenotype.

**Figure 4:**
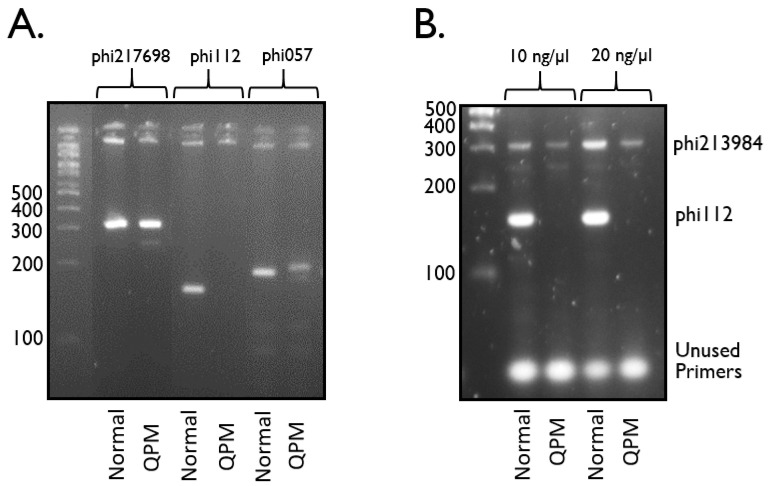
Standardization of primers and designing of multiplex PCR. (**A**). PCR using primers phi217698, phi112 and phi057. (**B**). Multiplex PCR using primers phi217698 and phi112. A total of 1 µL of two concentrations of genomic DNA, *viz*., 10 and 20 ng/µL, each of normal and QPM genomic DNA, were used in a 20 µL PCR for amplification.

To design an alternative protocol for determining the genetic makeup of the sample, a multiplex PCR was designed. Firstly, the normal maize and QPM were amplified using phi213984, phi112 and phi057 (Figure 4A). While phi213984 resulted in an identical amplicon of size 306 bp in both normal maize and QPM, phi112 amplified only the normal maize (size 155 bp), and phi057 resulted in size polymorphism (145 bp in normal maize and 165 bp in QPM). Then, both the phi213984 and phi112 were used together in multiplex format (Figure 4B). In normal maize, the phi213984 and phi112 resulted in respective products, whereas in QPM, only phi213984 resulted in the amplicon.

**Figure 5:**
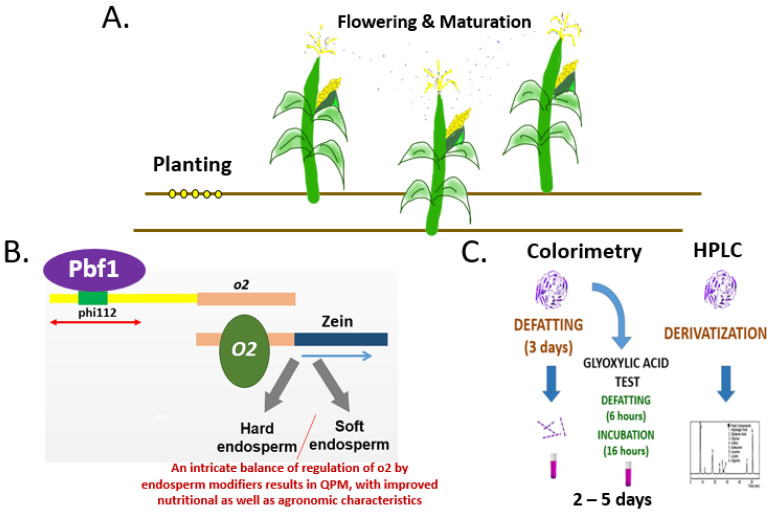
Schematic representation of the key stages and concerns in protein quality in maize. (**A**). Both the planting of authenticated seeds and maintenance of appropriate distance are necessary to ensure high protein quality in maize. (**B**). The authenticity of the planting material can be verified through the use of the dominant marker phi112 in a multiplex format for unambiguity. Pbf1 appears to master-regulate the high protein quality phenotype. (**C**). Analysis of the produced grain through available methods.

Given that protein quality is a commercial trait with immense financial implications, it is imperative to design standards and Quality Control parameters for high protein quality. As depicted in Figure 5A, two stages are critical in the case of maize. One is at the stage of planting, where it is important to ensure the absence of seed counterfeiting and that the QPM genotype is authentic. Due to the recessive nature of the trait, the authentication of seed at the genotype level is essential. Figure 5B provides a schematic representation of the models arising from information generated in the present work. PBF1 appears to be the master regulator for protein quality in maize by influencing the expression of the O2 transcription factor, which in turn is known to influence the expression of zein proteins and the overall nutritional status. In addition to this, the second critical stage is flowering. Pollen dispersal from nearby fields of normal maize can downgrade the quality of the presumed QPM produce. Therefore, it is necessary to check the quality of grain also. Figure 5C depicts the two commonly used tests, *viz*., colorimetry and high-performance liquid chromatography. These tests can take 2-5 days for laboratory analysis depending on different features of the protocol [46,47,48]. Recently, we designed a rapid protocol for differentiating normal maize from QPM at the grain stage. The developed protocol is under application for patent protection. This rapid protocol was successfully deployed to differentiate food products made from normal maize from those prepared from QPM [49]. The multiplex PCR assay based on the use of phi112 as a dominant marker for protein quality differentiation can be used as a supplement or a standalone test to screen for high protein quality material to enable differentiation of the bulk samples and aggregation of the QPM material. In case of any ambiguity in ascertaining the nature of the material being tested, its genetic makeup can be confirmed using the described PCR assay, in addition to its purity. Hence, the multiplex PCR for maize protein quality differentiation demonstrated here is a step towards ensuring higher remuneration for the QPM growers, which in turn would result in the supply of nutritious food products for health-aware consumers. The PCR assay, along with rapid protocols for grain-based testing can be incorporated into Standard Operating Protocols of farmers/Farmer Producer Organizations for end-to-end Quality Control of the QPM production chain. Given the well-defined protocols for conversion of elite germplasm to o2 and its subsequent development as QPM [50,51], it is expected that the market of biofortified maize will increase manifold in the coming times. SSR markers have been demonstrated to be a more cost-effective method than conventional methods for breeding, which, however, must be determined on a case-to-case basis [52]. The developed assay and the understanding of the mechanism reported herein will pave the way for further research in this direction.

## 4. Conclusions

The present study is the computational characterization of the phi112 marked DNA and its putative transcription factor protein. Based on the results obtained, the ‘recruiting’ function of PBF1 may include protein–protein interactions as well as transcription of the *o2* gene. The insight generated in this study can be taken forward to unravel the molecular network governing *opaque2* function and its exploitation for increasing maize’s essential amino acid content. Further, it will also help to envisage the possible use of gene editing techniques such as CRISPR-Cas9 to selectively modify the precise region to bring desirable changes in the phenotype without many associated undesirable effects. Given the documented benefits of QPM [53] and its potential market in the nutrition and health sector, it is imperative that enabling technologies are defined to allow its unambiguous differentiation for developing premium seed supply chains.

## Data Availability

Not Applicable.
